# Engineering conjugative CRISPR-Cas9 systems for the targeted control of enteric pathogens and antibiotic resistance

**DOI:** 10.1371/journal.pone.0291520

**Published:** 2023-09-12

**Authors:** Haiqing Sheng, Sarah Wu, Yansong Xue, Wei Zhao, Allan B. Caplan, Carolyn J. Hovde, Scott A. Minnich

**Affiliations:** 1 Animal, Veterinary and Food Science, University of Idaho, Moscow, Idaho, United States of America; 2 Department of Chemical and Biological Engineering, University of Idaho, Moscow, Idaho, United States of America; 3 Department of Plant Sciences, University of Idaho, Moscow, Idaho, United States of America; CINVESTAV-IPN, MEXICO

## Abstract

Pathogenic *Escherichia coli* and *Salmonella enterica* pose serious public health threats due to their ability to cause severe gastroenteritis and life-threatening sequela, particularly in young children. Moreover, the emergence and dissemination of antibiotic resistance in these bacteria have complicated control of infections. Alternative strategies that effectively target these enteric pathogens and negate or reduce the need of antibiotics are urgently needed. Such an alternative is the CRISPR-Cas9 system because it can generate sequence-specific lethal double stranded DNA breaks. In this study, two self-transmissible broad host range conjugative plasmids, pRK24 and pBP136, were engineered to deliver multiplexed CRSIPR-Cas9 systems that specifically target Enterohemorrhagic and Enteropathogenic strains of *E*. *coli* (EHEC and EPEC), *S*. *enterica*, and *bla*_CMY-2_ antibiotic resistance plasmids. Using *in vitro* mating assays, we show that the conjugative delivery of pRK24-CRISPR-Cas9 carrying guide RNAs to the EPEC/EHEC *eae* (intimin) gene can selectively kill enterohemorrhagic *E*. *coli* O157 *eae*^+^ cells (3 log kill at 6 h) but does not kill the isogenic *Δeae* mutant (P<0.001). Similar results were also obtained with a pBP136 derivative, pTF16, carrying multiplexed guide RNAs targeting *E*. *coli eae* and the *S*. *enterica ssaN* gene coding for the type III secretion ATPase. Another pBP136 derivative, TF18, carries guide RNAs targeting *S*. *enterica ssaN* and the antibiotic resistance gene, *bla*_CMY-2_, carried on the multi-drug resistant pAR06302. Introduction of pTF18 into bacteria harboring pAR06302 showed plasmids were cured at an efficiency of 53% (P<0.05). Using a murine neonate EPEC infection model, pTF16 was delivered by a murine derived *E*. *coli* strain to EPEC infected mice and showed significant reductions of intestinal EPEC (P<0.05). These results suggest that establishing conjugative CRISPR-Cas9 antimicrobials in the intestinal microbiome may provide protection from enteric pathogens and reduce antibiotic resistance without disrupting the normal microbiota.

## Introduction

Pathogenic *Escherichia coli* and *Salmonella enterica* represent a continuous worldwide public health threat due to their abilities to elicit severe gastroenteritis and life-threatening complications in humans, especially infections of the very young or elderly [1–3]. Although the use of antibiotics is successful in treating acute infections, their misuse has led to the spread of antibiotic resistance, which is now a global health emergency [4,5]. Moreover, antibiotic treatment reduces the number, and disrupts normal physiological activities, of the gastrointestinal microbiota. This dysbiosis can have profound effects on human and animal health [6,7], especially in children [8,9]. Therefore, new strategies that selectively eradicate or reduce pathogens and antibiotic resistance genes (ARG) without disrupting the overall beneficial microbiota, are needed.

CRISPR-Cas systems are protective barriers in prokaryotes that function in the adaptive immunity to recognize and destroy invading plasmids and viruses [10]. The well-studied type II CRISPR-Cas9 system consists of two components: the Cas9 nuclease protein and a guide RNA (gRNA). Cas9 binds to gRNAs to form a complex that scans the target locus containing a protospacer adjacent motif (PAM) sequence 5’-NGG-3’. Once a PAM is recognized, the DNA of the target base-pairs with the gRNA, and the nuclease activity of Cas9 creates a double-stranded break [11]. The CRISPR-Cas9 system has been adapted for a variety of genetic engineering uses including genome-wide editing in both prokaryotics and eukaryotics [12]. In bacteria, introduction of gRNA-directed Cas9 double-stranded breaks in the chromosome collapses replication forks leading to cell death. When directed against plasmid DNA, plasmids are eliminated. Therefore, the CRISPR-Cas9 system is a potential attractive alternative to treat bacterial infections [13,14]. Past studies demonstrate the promising results of CRISPR-Cas9 systems as antimicrobials for selective eliminations of bacteria [15–18] and antibiotic resistance gene-carrying plasmids [19–23]. Since Cas9 is solely dependent on gRNAs for DNA sequence identification and cutting, CRISPR-Cas9 systems can be engineered with an array of gRNAs to target multiple independent DNA sequences [24,25]. Such array complexes allow simultaneous control of a broad-spectrum of bacterial species by targeting common conserved regions of shared virulence genes and/or antibiotic resistance gene-carrying plasmids. However, delivery of CRSIPR-Cas9 system remains a major hurdle for these applications. The success of CRISPR-Cas9 antimicrobials depends on their efficient delivery to the target population.

Conjugation is a natural efficient and fast mechanism for genetic transfer. It allows single-event delivery of large DNA fragments from a donor to a recipient bacterium with high fidelity and speed. The speed of DNA transfer is illustrated by *E*. *coli* Hfr strains, which have the conjugative F factor (plasmid) integrated into the *E*. *coli* chromosome of 4.6 M basepairs. It only takes ~100 min for an Hfr strain to transfer the entire *E*. *coli* genome to a recipient cell. This is a rate of ~46,000 base pairs/min. Because most self-transmissible conjugative plasmids are in the range of 40 kbp to 100 kbp, their independent transfer easily occurs in 1–2 m [26].

Self-transmissible conjugative plasmids generally replicate independently of the host genome and encode the entire conjugative machinery that facilitates their own transfer from one bacterial strain or species to another [27]. The conjugal machinery comprises three essential components: the relaxosome, the coupling protein, and a type IV protein secretion system. Importantly, the donor cell, during a mating with a recipient, maintains a copy of the plasmid it transfers thus, allowing the exponential expansion of a plasmid in the recipient population. This is the reason that conjugative plasmids play a critical and major role in the spread of antibiotic resistance in the microbiota and environment [28,29].

Over the past two decades, increasing attention has been paid to *bla*_CMY-2_ plasmids [30,31]. These IncA/C plasmids carry multiple antibiotic resistance genes including *bla*_CMY-2_, which encodes an AmpC-type beta-lactamase that hydrolyzes third generation cephalosporins. The IncA/C plasmids have been disseminated within and between several important enteric pathogens, including *E*. *coli*, *S*. *enterica*, *Klebsiella pneumoniae*, and *Yersinia pestis*, recovered from humans, fish, and farm animals [31]. Thus, conjugation and CRISPR are both natural mechanisms related to genetic transfer, but with opposite functions. Given that bacterial conjugation is responsible for antibiotic resistance dissemination, harnessing it to deliver CRISPR-Cas9 antimicrobials against a target is an attractive “fight-fire-with-fire” strategy. Engineering self-transmissible conjugative plasmids of the incompatibility subgroup IncP-1, *e*.*g*. pRK24 [32,33] and pBP136 [34] with specific CRISPR-Cas9 systems takes advantage of this highly efficient approach. These plasmids are efficiently spread and maintained in a broad range of recipient enteric bacterial genera.

In the present study, we engineered pRK24 and pBP136kan by introducing Cas9 and gRNAs specific for a highly conserved signature sequence of the *eae* (intimin) genes of EPEC and EHEC, a highly conserved sequence of *ssaN* genes (type III secretion) of *S*. *enterica*, and of a conserved sequence in the plasmid-borne antibiotic resistance-*bla*_CMY-2_ gene. The effects of conjugative CRISPR-Cas9 antimicrobials on the selective eliminations of the bacterial pathogens and the antibiotic resistance gene-carrying plasmids were investigated. Experiments included (i) engineering pRK24-based plasmid pRK24-CRISPR, and testing the efficiency of conjugative delivery of the CRISPR-Cas9 system and its ability to selectively kill EHEC O157:H7 via *in vitro* mating assays, (ii) engineering pBP136-based plasmids pTF16 and pTF18 with CRISPR systems and testing their abilities to deliver the multiplexed CRISPR-Cas9 arrays into bacteria for selective and simultaneous eliminations of the targeted pathogens of EPEC, EHEC, *Salmonella* and the *bla*_CMY-2_ plasmids *in vitro*, and (iii) testing pTF16 for its ability to limit EPEC O55:H6 carriage in a neonate mouse model.

## Results

### Engineering self-transmissible conjugative plasmids to deliver CRISPR-Cas9 systems targeting EPEC, EHEC, *S*. *enterica* ser. Typhimurium, and IncA/C *bla*_CMY-2_ plasmids

**Fig 1:**
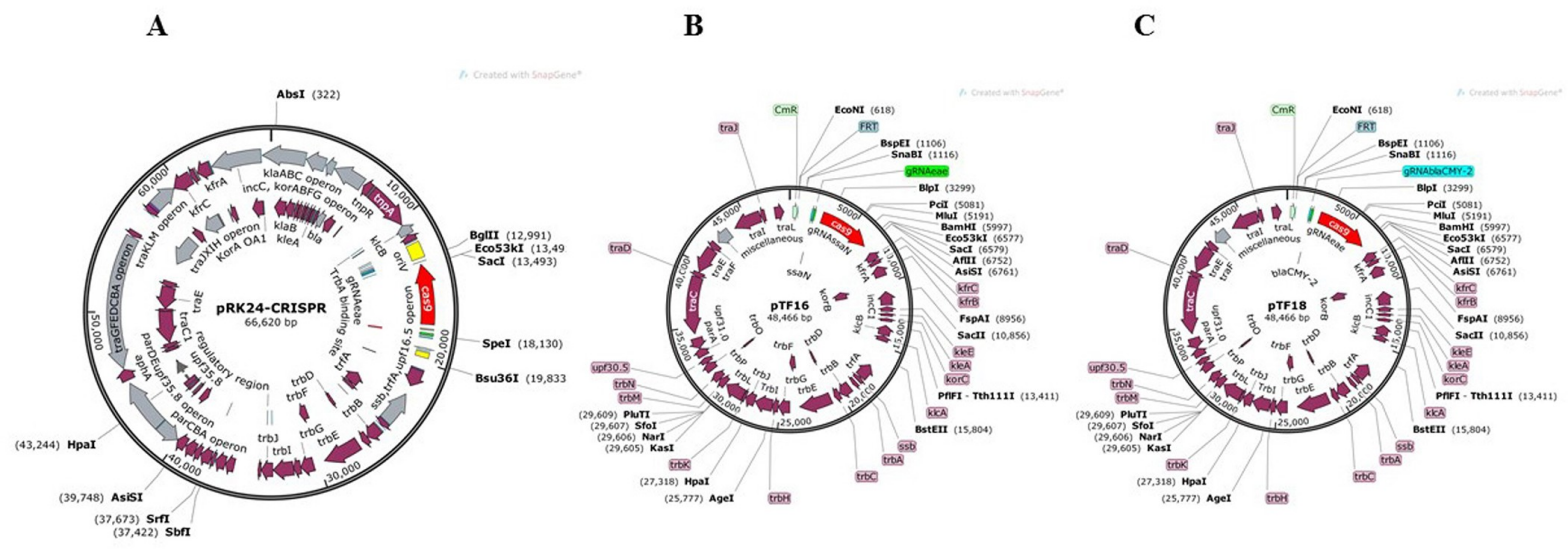
Maps of the self-transmissible conjugative plasmids carrying CRISPR-Cas9 antimicrobials. (A) Plasmid pRK24-CRISPR was engineered to carry Cas9 and gRNA*eae* targeting *eae*-positive pathogenic *E*. *coli*. (B) Plasmid pTF16, a derivative of pBP136, contains Cas9 and dual gRNAs (gRNA*eae* and gRNA*ssaN*) targeting pathogenic *E*. *coli* (EHEC and EPEC) and *S*. *enterica*. (C) Plasmid pTF18, a derivative of pBP136, contains Cas9 and dual gRNAs (gRNA*ssaN* and gRNA gRNA*bla*_CMY-2_) that targeting *S*. *enterica* and *bla*_CMY-2_ plasmids.

To use CRISPR-Cas9 as an antimicrobial against EPEC and EHEC, we chose *eae* as the target. The “attaching and effacing” lesion (A/E) is a common mechanism of intestinal colonization shared by the two pathogenic *E*. *coli* groups [35], and *eae*, which encodes intimin, is an essential virulence factor for both. Bioinformatic analysis identified a 20-nt *eae* invariant sequence conserved among all the *eae* subtypes of EPEC and EHEC serogroups. The *de novo* synthesized gRNA*eae* containing a constitutive promoter pJ23119, the 20-nt targeting sequence, and *cas9* with its native *S*. *pyogenes* promoter were used to construct pRK24-CRISPR as described in Materials and Methods (Figs 1A and S1).

To target *Salmonella*, we selected the type III secretion system *ssaN* gene that encodes a *Salmonella-*specific ATPase required for virulence [36]. gRNA*ssaN* containing a 20-nt conserved targeting sequence was synthesized *de novo*. Similarly, a gRNA*bla*_CMY-2_ was synthesized using a 20-nt conserved targeting sequence for *bla*_CMY-2_ plasmids. We engineered pBP136Kan to express Cas9 with its native *S*. *pyogenes* promoter and gRNA*ssaN* under the control of the pJ23119 constitutive promoter (Fig 1B and 1C). The resulting engineered self-transmissible conjugative plasmids pTF16 and pTF18 carry functional CRISPR systems that target either *eae* and *ssaN* or *eae* and *bla*_CMY-2,_ respectively (Fig 1 and Table 4). In addition, a *cat* gene flanked by FRT repeats was included in the plasmids to replace Kan of pBP136kan for detection. Therefore, by a simple electroporation of pCP20 [37], the selective marker gene on the engineered plasmids can be removed to avoid spread of the antibiotic resistance trait for practical implementations of these conjugative CRISPR-Cas9 systems in the future.

### pRK24-CRISPR, expressing gRNA*eae* and Cas9, selectively kills EHEC O157:H7 *eae*^+^/*Δtir* cells, but not *Δeae/tir*^*+*^ EHEC O157:H7 cells

**Fig 2:**
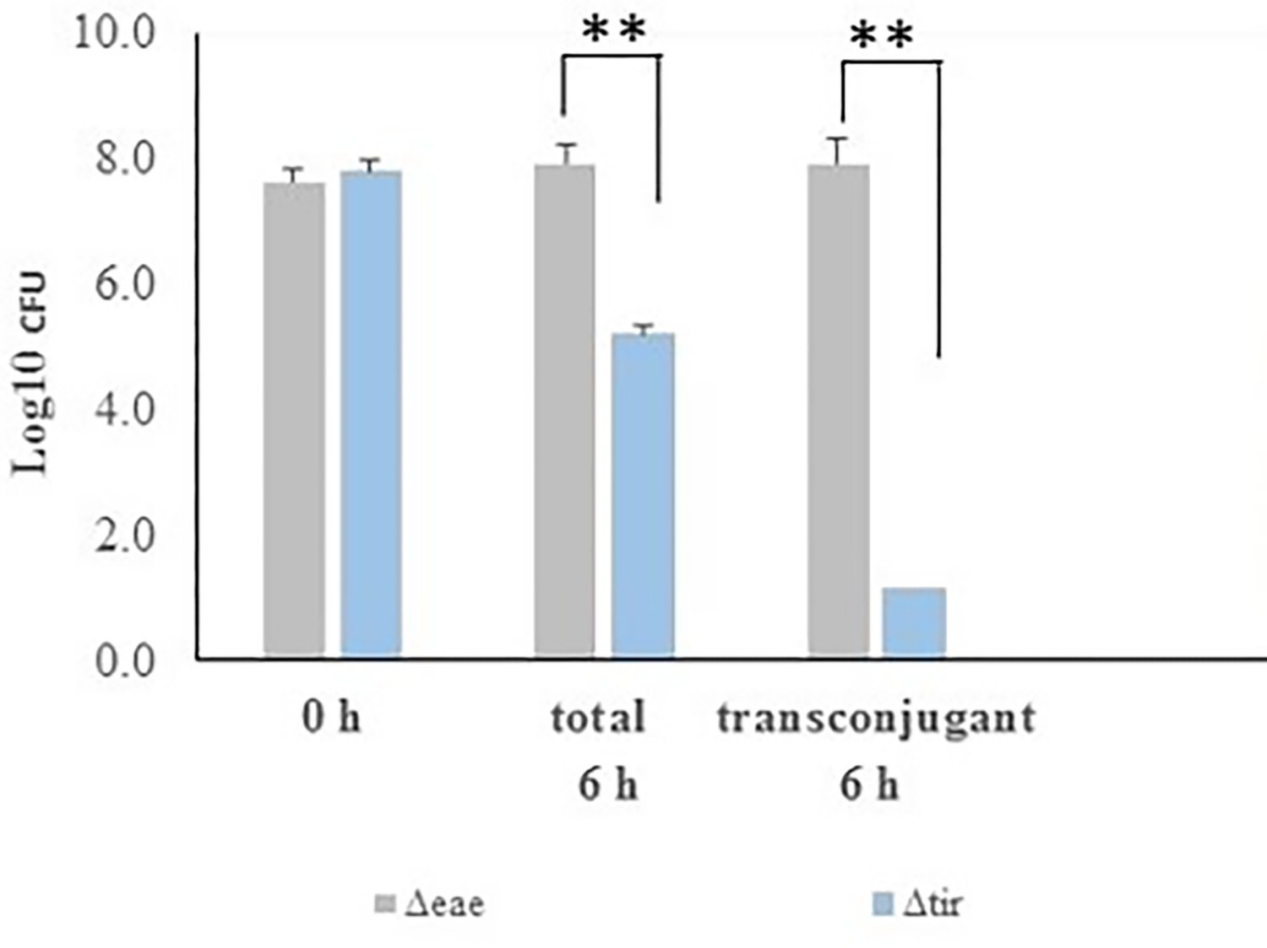
Conjugation with pRK24-CRISPR kills *eae*^+^ and not *Δeae E*. *coli* O157:H7. A triparental mating assay was performed to evaluate the effect of pRK24-CRISPR conjugation to the targeted O157:H7 strain 905*Δtir* and the non-targeted strain 905*Δeae*. The numbers of the recipient bacteria and the transconjugants were enumerated following 6 h mating done in triplicate. Error bars represent the geometric mean log values of bacterial counts plus the geometric standard deviation. ** P<0.001 by the Student’s t-test.

We assessed the efficacy of conjugative delivery of gRNA*eae* and Cas9 via pRK24-CRISPR by its ability to selectively kill the targeted EHEC strain. In a triparental mating assay, *E*. *coli* K12 was used as the donor of pRK24-CRISPR (Rif^R^, Cm^R^), and two isogenic mutants of EHEC O157:H7, 905 *eae*^+^/*Δtir* (Nal^R^) and 905*Δeae/tir*^*+*^ (Kan^R^) as recipients. Because both *eae* and *tir* are chromosomally located within the locus of enterocyte effacement, 905 *eae*^*+*^*/Δtir* represents the gRNA*eae* target while 905*Δeae/tir*^*+*^, lacking the gRNA*eae* target, represents the negative control. Following a six h mating, the numbers of the recipient bacteria and the transconjugants were enumerated. We observed 96% of 905*Δeae/tir*^*+*^ control recipient cells acquired pRK24-CRISPR (Cm^R^Nal^R^) from the *E*. *coli* K12 donor with no lethal effect (Fig 2), in fact the number of 905*Δeae/tir+* cells increased during the six h mating (Fig 2). These results showed an efficient rate of conjugative plasmid transfer. In contrast, 905 *eae*^*+*^*/Δtir* cells (Nal^R^) showed a 99.9% (3 log) reduction in numbers. These results showed an efficient rate of plasmid transfer, as with control cells, but also the sequence-specific killing due to conjugative delivery of the pRK24-CRISPR-Cas9 (Fig 2). pRK24 has high conjugation efficiency in EPEC and EHEC strains and can be stably maintained in these strains. However, pRK24 has relative lower conjugation efficacy in other *Enterobacteriaceae* species such as *Salmonella* (< 10^−3^). In addition, it contains multiple ARGs that complicate the work of CRISPR engineering and future applications.

### Conjugation of pTF16 selectively and simultaneously kills the targeted pathogenic *E*. *coli* and *Salmonella enterica ser* Typhimurium strains

pBP136-based plasmids have high conjugation frequencies in both *E*. *coli* and *Salmonella*. To evaluate the ability of its derivative pTF16 to deliver the CRISPR-Cas9 antimicrobials against multiple serogroups of EPEC, EHEC and *S*. *enterica* ser Typhimurium, separate mating assays were performed using *E*. *coli* EC100 containing pTF16 as the donor (Cm^R^) and *E*. *coli* K12 (Rif^R^), EPEC O26:H11, O55:H6, O128:H2 (all Nal^R^), EHEC O157:H7 (Nal^R^), *Salmonella typhimurium* TR5877 (Rif^R^) and ATCC 14028 (Nal^R^) as recipients. We observed the significant killing of all targeted strains, with cell deaths ranging from 91 to 96%. There was no killing of the *E*. *coli* K12 control strain which acquired pTF16. In fact, the total *E*. *coli* K12 cell numbers increased during the six h mating period (Table 1).

**Table 1:** Mating killing assay of pTF16*.

Recipient	Killing efficiency (%)
*E*. *coli* K12 EPEC	-75.64 (±4.34)
O55:H6	95.71 (±2.27)
O128:H2	95.05 (±2.13)
O26:H11	91.08 (±5.38)
EHEC	
O157:H7 ATCC 43894	96.23 (±1.87)
*S*. *enterica* ser Typhimurium	
TR5877	94.53 (±3.02)
ATCC 14028	95.89 (±2.15)

*EC100 harboring pTF16 as the donor mating with each strain for six h. Percent killing efficiency was calculated from triplicate experiments using the equation (1-remaining number of the recipient cells / the initial number of the recipient cells) × 100%.

**Fig 3:**
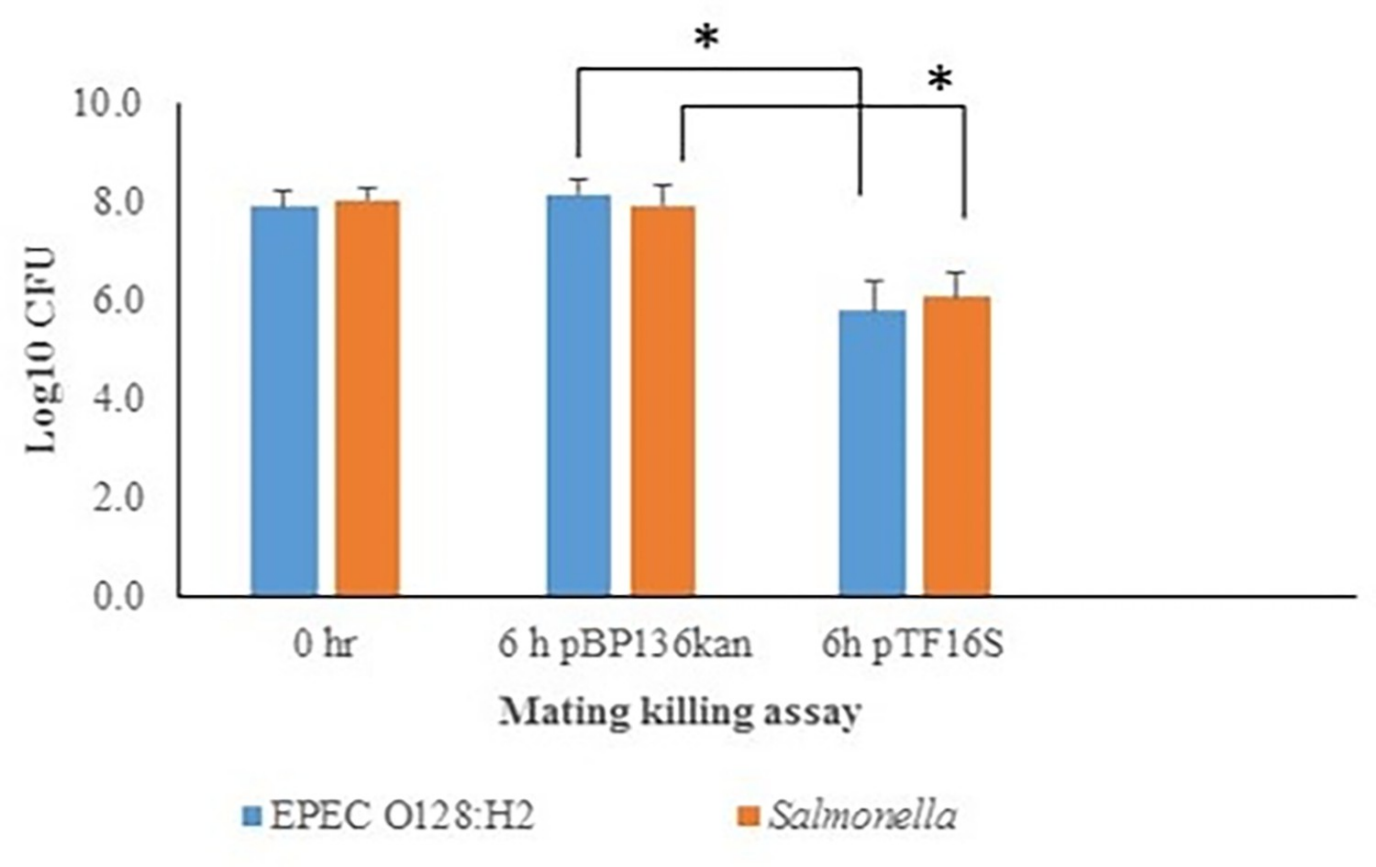
Conjugation of pTF16 simultaneously kills EPEC and *S*. *enterica*. In the triparental mating assays, EPEC O128:H2 (Nal^R^) and *S*. *enterica* ser Typhimurium TR5877 Rif^R^) mated with EC100 (Cm^R^) strains harboring pBP136kan or pTF16. The numbers of the recipient cells were enumerated after 6 h mating. Error bars represent the geometric mean of log values of bacterial counts plus the geometric standard deviation. * P < 0.001.

**Fig 4:**
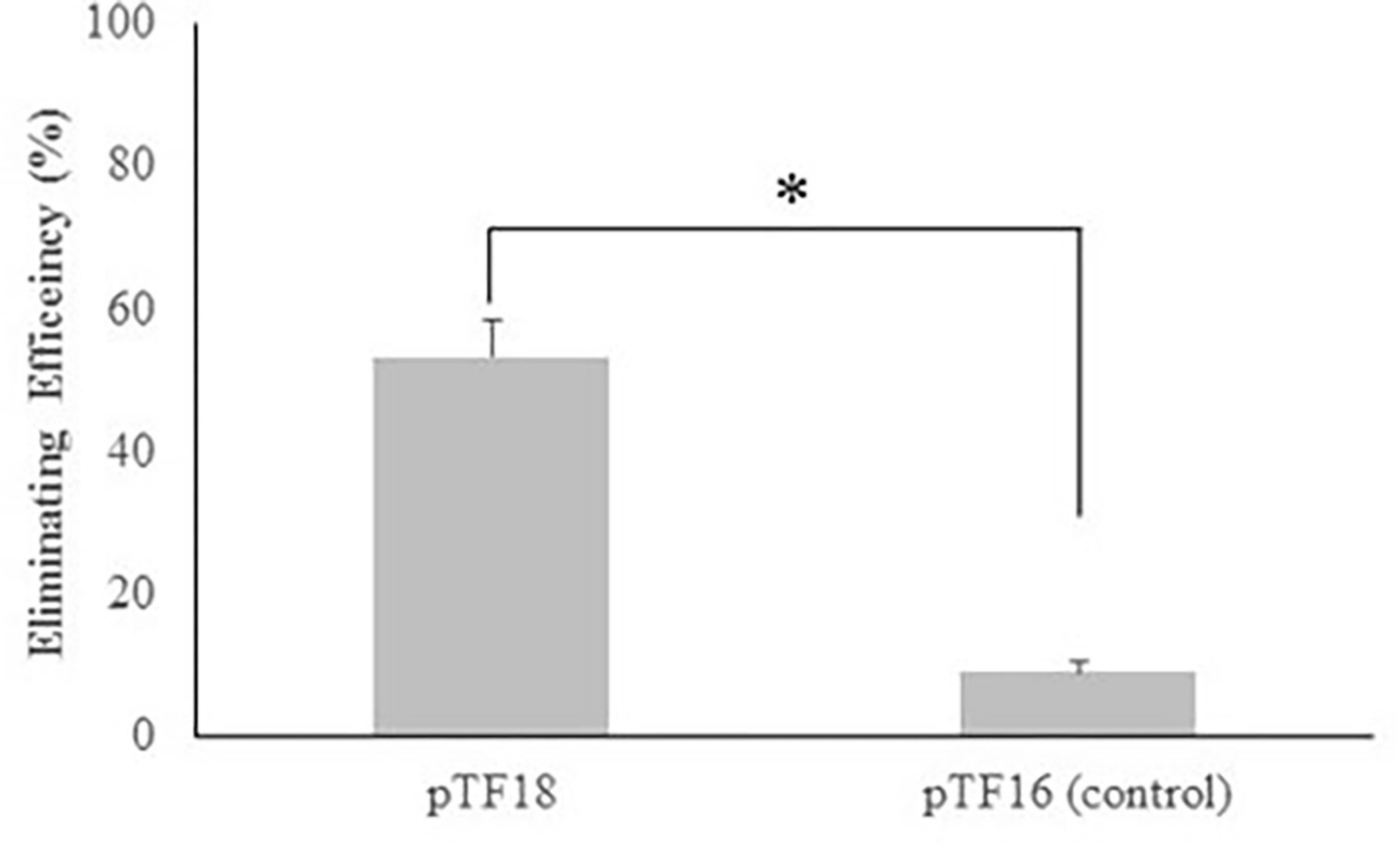
Conjugation of pTF18 eliminates *bla*_CMY-_2 plasmids from the host *E*. *coli*. *E*. *coli* C3b harboring pAR60302 (Amp^R^, Cm^R^) was mating with EC100 pTF18 or pTF16. Elimination efficiency is the percentage of the recipient cells that lost Amp resistance following the mating. Data points represent the mean value plus the geometric standard deviation. * p<0.05.

Next, a triparental mating assay was performed to evaluate the ability of pTF16 conjugation to simultaneously kill *E*. *coli* and *S*. *enterica* strains. We used EC100 pTF16S as the donor, in which the built-in Cm^R^ cassette was removed by transforming EC100 pTF16S with pCP20 [36]. EPEC O128:H2 (Nal^R^) and *Salmonella enterica* ATCC 14028 (Rif^R^) were used as recipients. In a parallel control mating, EC100 pBP136kan (Kan^R^) served as the donor (Cm^R^) to mate with both recipients. Following a six h mating, the numbers of the *E*. *coli* and *S*. *enterica* ATCC14028 were enumerated. The numbers of EPEC O128:H2 and *S*. *enterica* ATCC 14028 show 2.13 and 1.78 log reductions, respectively. In contrast, the numbers of the EC100 pBP136kan control recipients were unchanged or slightly increased after the six h mating (Fig 3). It is notable that pTF16S, which has no selective marker gene due to the removal of the Cm^R^ cassette, showed no difference in conjugation or killing efficiency compared with pTF16 (Table 1 and Fig 4).

### Conjugation of pTF18 eliminates *bla*_CMY-2_ plasmids from *E*. *coli*

To access the ability of the CRISPR-Cas9 systems to target ARG-carrying plasmids, pTF18 containing gRNA*bla*_CMY-2_ was engineered. The IncA/C plasmid pAR06302 harboring *bla*_CMY-2_ was used as a target. Donor EC100 pTF18 (Cm^R^) was mated overnight with recipient *E*. *coli* C3b pAR06302 (Nal^R^, Amp^R^, Cm^R^). As a control, the above recipient cells were mated overnight with EC100 containing pTF16, which lacks gRNA*bla*_CMY-2_. The results showed that 53.3% *E*. *coli* C3b cells became sensitive to Amp due to loss of *bla*_CMY-2_ while only 9% of *E*. *coli* C3b cells were Amp^S^ after mating with the control EC100 pTF16 (Figs 4 and S1). Colony-PCR was performed to detect the *cas9*-gRNA*bla*_CMY-2_ cassette in the Amp^S^ colonies derived from both pTF18 and pTF16 matings. The results showed that more than 85% of Amp^S^ colonies from the mating with the pTF18 donor were *cas9*-gRNA*bla*_CMY-2_ positive indicating these cells were transconjugants. In contrast, most of the Amp^S^ colonies from the control mating lacked plasmid pTF16. The 9% of *E*. *coli* C3b that lost pAR06302 during the overnight co-culturing with EC100 pTF16 might reflect the fitness cost of maintaining pAR06302 in the absence of selection, as reported previously [38,39]. Together, these results showed that conjugative delivery of CRISPR antimicrobials selectively killed the targeted bacterial pathogens and cured ARG-carrying plasmids *in vitro*.

### CRISPR-Cas9 antimicrobial delivered by pTF16 reduces the burden of EPEC in the murine intestine

**Fig 5:**
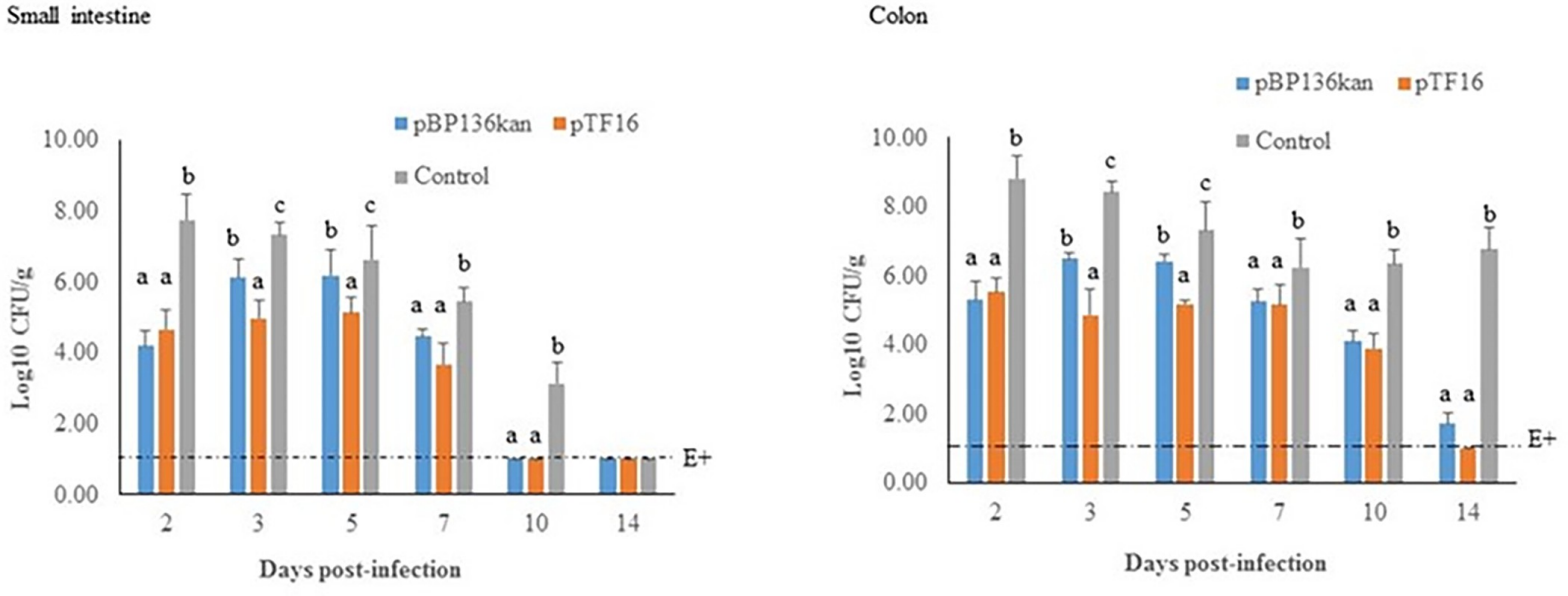
CRISPR-Cas9 antimicrobial delivered by pTF16 reduces the burden of EPEC O55:H6 in the murine intestine. Neonate mice were orally infected with 10^5^ CFU EPEC. On day 1 and 2 post-infection, the pups in three groups were treated with *E*. *coli* MF1 pBP136kan, *E*. *coli* MF1 pTF16, or PBS, respectively. Small intestine and colon tissues were collected, homogenized and plated for bacterial counts. E+ indicates samples positive for EPEC O55:H6 only by an enrichment procedure. Error bars represent the geometric mean plus the geometric standard deviation. Significance is shown via grouping: a = significantly different from groups marked with b or c; b = significantly different from groups marked with a or c; c = significantly different from groups marked with a or b. * p ≤ 0.05.

Because pTF16 selectively and efficiently killed EPEC O55:H6 in filter mating experiments, we evaluated its efficacy for the reduction/elimination of EPEC from mammalian intestines using a murine neonate model of EPEC colonization [40]. Three groups of 3-day old mice were orally infected with 10^5^ CFU of EPEC O55:H6 (Nal^R^). On day 1 and 2 post-infection (PI), the Group 1 pups were treated with *E*. *coli* MF1 pBP136kan (Rif^R^, Kan^R^), Group 2 were treated with *E*. *coli* MF1 pTF16 (Rif^R^, Cm^R^), and Group 3, received only PBS. (Table 2). On days 2, 3, 5, 7, 10, and 14 PI, the small intestine and colon from at least 2 animals in each group were collected, homogenized in PBS, diluted, and plated on LB agar plates containing the appropriate antibiotics for bacterial counts. We observed that oral administration of EPEC O55:H6 with 10^5^ CFU/mouse produced a stable infection for more than two weeks. During this time the numbers of O55:H6 recovered from the intestines ranged from 10^6^ to 10^8^ CFU per gram tissue (Fig 5). Consistent with the previous study [40], we did not observe clinical symptoms such as watery diarrhea in infected neonate mice.

**Table 2:** Mouse experiment.

Age (d) /Infection +Treatment	Group 1 n = 14	Group 2 n = 15	Group 3 n = 14
3	EPEC	EPEC	EPEC
4	MF1 pBP136kan	MF1 pTF16	PBS
5	MF1 pBP136kan	MF1 pTF16	PBS

Note: MF1, a murine commensal *E*. *coli*; pBP136kan, a control conjugative plasmid; pTF16, an engineered pBP136kan derivative with insertion of the CRISPR-Cas9 targeting EPEC; Infection: 10^5^ CFU EPEC O55:H6 in 1 μl PBS. Treatment: 10^7^ CFU in 10 μl 20% sucrose PBS per pup.

The conjugative transfer of pBP136kan in the GI tracts of mice in Group 1 was monitored. The transconjugants of O55:H6 (Kan^R^ and Nal^R^) recovered from the tissues ranged from 10^1^ to 10^4^ CFU/gram. We observed the efficiency of conjugation of pBP136kan in the mouse intestine was lower (<10^−2^) compared with that *in vitro* (>10^−1^). The total transconjugants of the intestinal microbiota could not be monitored due to lack of total recovery methods. Comparing Group 1 and 2, the numbers of EPEC O55:H6 recovered from the mice of MF1 pTF16 treatment were lower than those of MF1 pBP136kan treatment on every sampling day except day 2 PI. There were significantly lower numbers of EPEC O55:H6 in the mice of MF1 pTF16 treated Group 2 on day 3 and 5, compared with those of MF1 pBP136kan treated Group 1. The data suggest that the CRISPR-Cas9 antimicrobial delivered by conjugative plasmid reduced the burden of EPEC in the murine intestine. Strikingly, significant reduction of EPEC O55:H6 loads in the small intestines and colons of the mice in Group 1 or Group 2 was observed on every sampling day, except day 14 on the sample of small intestine, as compared with those mice treated with PBS in Group 3 (Fig 5). The *in vitro* mating assay indicated that MF1 pBP136kan did not affect the numbers of EPEC O55 following 6 h or overnight mating periods, while both EC100 and MF1 harboring pTF16 efficiently killed EPEC O55 in the mating assays (Table 1). Therefore, the significant reduction of EPEC O55 in MF1 pBP136kan treated Group 1, compared with the PBS control Group 3, may be attributed to an antagonistic effect or competitive exclusion of EPEC O55:H6 exerted by *E*. *coli* MF1 in the intestines of the mice, which was a murine commensal isolate from one of the pregnant mothers.

## Discussion

CRISPR-Cas9 antimicrobials represent a promising alternative to combat bacterial infections and drug resistance because of the accuracy, specificity, modularity, and versatility. However, the application has been restricted by the challenge of their delivery to the target population. Over the past decade, CRISPR-Cas9 antimicrobials have been incorporated into phages, phagemids, or transformable plasmids for the recognition of and delivery to target cells. These delivery systems have several limitations: 1) phagemids are replication-deficient, therefore, a very large number of phagemids and repetitive treatments would be required to be effective *in vivo*; 2) phages generally have a narrow bacterial host range; 3) the rise of phage resistance through receptor mutations is a major problem of phage treatment; 4) phage DNA can be degraded by bacterial hosts by restriction modification systems; 5) systemic side-effects may be caused by endotoxin exposure in the intestine, both from the bacteriophage preparation itself and resulting bacterial lysis; 6) transformable plasmids are not self-transmissible and cannot be maintained in the absence of selection [12,14,16,17]. To overcome these hurdles of the aforenamed systems, we present in this study a different strategy by harnessing and engineering self-transmissible conjugative plasmids for the delivery of CRISPR-Cas9 antimicrobials.

Self-transmissible conjugative plasmids of the IncP-1 group have a broad host range and can be stably maintained in almost all Gram-negative bacteria, some of them have also been demonstrated to conjugate to Gram-positive bacteria [41]. Therefore, we engineered IncP-1α pRK24 and IncP-1β pBP136 with specific CRISPR-Cas9 systems targeting pathogenic *E*. *coli*, *Salmonella*, and *bla*_CMY-2_-specific anti-microbial resistance. pBP136 is particularly desirable for CRISPR-Cas9 engineering and for *in vivo* applications due to its smaller size and lack of accessory mobile and antibiotic elements [42]. We showed that the conjugation of the CRISPR-Cas9-engineered plasmids selectively and simultaneously killed a broad spectrum of targeted pathogens regardless of the serotypes, and efficiently removed the *bla*_CMY-2_ plasmids from the bacterial population *in vitro*, as well.

Pathogenic *E*. *coli* and *S*. *enterica* are enteric pathogens and are particularly troublesome when the bacteria acquire ARGs. We applied the CRISPR-Cas9 antimicrobial for the control of intestinal EPEC *in vivo*. In a murine model of EPEC colonization, the murine *E*. *coli* MF1 strain was used as the donor for the conjugative delivery of pTF16 to intestinal EPEC O55:H6. Although *E*. *coli* MF1 pTF16 significantly reduced the bacterial load at the height of infection, there was no difference from the controls on the later sampling days. There are two possible reasons for these results: 1) the intestinal conjugation frequency of pBP136kan plasmid is suboptimal (<10^−2^) compared with that *in vitro* (>10^−1^); 2) *E*. *coli* MF1, which was isolated from a pregnant female, may have an antagonistic or a competitive exclusion effect in the infant mice. Thus, the administration of *E*. *coli* MF1 may have served as a probiotic and provided protection against EPEC O55:H6 infection of the neonate mice. This could mask the contribution of the CRISPR antimicrobial to the reduction of EPEC during the experiment period. An alternative and attractive option could be to use the *E*. *coli* MF1 CRISPR-Cas9 gRNA*eae* as a probiotic in a mouse and then challenge with EPEC. In this approach, the conjugative plasmid would presumably be amplified in the Gram-negative murine population increasing the chances of destroying EPEC before an active infection can be established.

Bacterial conjugation is contact-dependent. Various factors such as oxygen levels, nutrient availability, colonization niches, hosts, mating-pair stabilization mechanism, and the composition and density of intestinal microbiota contribute to the transfer efficiency of CRISPR antimicrobials to targeted populations [43]. Mammalian intestines serve as a genetic melting pot for horizontal gene transfer. Certain features in the intestinal environment provide favorable conditions for plasmid conjugative transfer. The intestinal microbial population is extremely diverse and dense, with over 10^10^ microorganisms per milligram of content. The immediate physical proximity and wide range of neighboring cells create an ideal environment for bacterial conjugation [44,45]. A growing number of studies using mammalian models to investigate bacterial intestinal gene transfer show a generally higher rate of transfer for some plasmids *in vivo* than *in vitro* laboratory conditions [46–52]. This indicates existence of indigenous transfer promoting factors in the intestine. Interestingly, intestinal inflammation boosts horizontal gene transfer between pathogenic and commensal *Enterobacteriaceae* [53]. Understanding how conjugative plasmids behave within the complex gastrointestinal tract is a critical step towards developing and improving the conjugative system for the delivery of CRISPR-Cas9 antimicrobials.

The CRISPR-Cas9 system offers not only specificity but also a broad-spectrum in targeting specific DNA sequences. For antimicrobial applications, a multiplexing design of gRNAs allows the targeting of multiple specific DNA sequences across bacterial species and genera. Furthermore, the conjugative delivery allows the transfer of the programmed CRISPR-Cas9 systems among related and unrelated bacteria. Bacterial donor cells carrying CRISPR-engineered self-transmissible plasmids could be used as probiotics to provide broad-spectrum immunity against enteric pathogens and ARGs. To our knowledge, this is the first report on delivery of CRISPR-Cas9 antimicrobials by the engineered self-transmissible conjugative plasmids to control the targeted enteric pathogens and to block the spread of drug resistance. It is envisaged that this approach could be optimized for therapeutic application, or for removing enteric pathogens and antibiotic resistance from the microbiota of humans and farm animals. Controlling EHEC in its ‘silent’ ruminant reservoirs and thus preventing contamination of food is one obvious goal of this work.

## Materials and methods

### Bacterial strains, plasmids, growth conditions

Bacterial strains and plasmids used in this study are listed in Table 3. Strains were grown on plates or in liquid Luria broth Miller (LB) medium at 37°C; in the case of liquid medium, the cultures were grown with aeration (180 rpm). *E*. *coli* strains EC100 (Lucigen Corp., Middleton, WI), TOP10 (Invitrogen Themo Fisher) or cc118 [54] were used for routine plasmid propagation. When appropriate, antibiotics were added to the growth medium (Ampicillin, Amp, 100 μg/ml; nalidixic acid, Nal, 25 μg/ml; rifampin, Rif, 30 μg/ml; kanamycin, Kan, 50 μg/ml; chloramphenicol, Cm, 50 μg/ml; tetracycline, Tet, 30 μg/ml; Sigma-Aldrich, St. Louis, MO).

**Table 3:** Bacterial strains and plasmids.

Strains	Relevant genotype and phenotype features	Reference/Source
EC100	*E*. *coli* cloning host, providing repA in trans. F-, *araD*139 (*ara ABC*-leu)7679, *galU*, *galK*, *lacX*74, *rspL*, *thi*, *repA* of pWV01 in *glgB*, km	Lucigen
cc118	r^-^ m^-^ *λpir*^+^; cloning strain	[55]
S17-1	*pro recA thi hsdR* Hfr RP4-2 (Tc::Mu) (Km::Tn7) Sm^R^ Tp^R^, *λ*pir lysogen	[54]
MG1655	*E*. *coli* K12 strain, Rif^R^	[56]
O157:H7 43894	clinical isolate, *stx1*^+^/*stx*2^+^, Nal^R^	ATCC
O157:H7 905	clinical isolate, *stx1*^-^/*stx2*^+^	[56]
905*Δeae*	EEHC 905 with *eae* deletion, Kan^R^	[57]
905*Δtir*	EHEC 905 with *tir* deletion, Nal^R^	[57]
EPEC O26:H11	clinical isolate, *eae*^*+*^, Nal^R^	Dr. Besser
EPEC O55:H6	clinical isolate, *eae*^*+*^, Nal^R^	Dr. Besser
EPEC O128:H2	clinical isolate, *eae*^*+*^, Nal^R^	Dr. Besser
*E*. *coli* C3b	*E*. *coli* DH5α carrying plasmid pAR06302-*bla*_CMY-2_	[30]
*Salmonella* TR5877	*Salmonella typhimurium*, Rif^R^	ATCC
*Salmonella* 14028	*Salmonella typhimurium*, Nal^R^	ATCC
**Plasmids**		
pRK24	Broad-host range self-transmissible conjugative plasmid, Tet^R^, Amp^R^	Addgene, [42]
pBP136kan	Derivative of a broad-host range self-transmissible conjugative plasmid pBP136, Kan^R^	[34]
pUC57	Cloning vector, Amp^R^	GenScript
pKD3	Template plasmid, Amp^R^, Cm^R^	[37]
pCas9	S. pyogenes *cas9* cloned in pACYC184, Cm^R^	Addgene
pCP20	Temperature-sensitive replicon, thermal induction of FLP synthesis, Amp^R^, Cm^R^	[37]
pWS10	Amp^R^	This work
pWS20	Cm^R^	This work
pWS20tet	pWS20 with pWS20 with insertion of a *tet* fragment of pRK24, Cm^R^	This work
pWS21	pWS20 with gRNA*ssaN* insertion; Cm^R^	This work
pWS21B	pWS20 with gRNA*bla*_CMY-2_ insertion, Cm^R^	This work
pRK24-CRISPR	pRK24 derivative with CRISPR targeting *eae*; Cm^R^, Amp^R^	This work
pTF16	pBP136kan derivative with CRISPR targeting *eae* and *ssaN*, Cm^R^	This work
pTF16S	pTF16 with Cm marker removed, Cm^S^. Kan^S^	This work
pTF18	pBP136kan derivative with CRISPR targeting *eae* and *bla*_CMY-2_, Cm^R^	This work

### Molecular biology techniques, gRNA designs, and plasmid constructions

PCR was performed using Q5 DNA polymerase (New England Biolabs (NEB), Ipswich, MA) for molecular cloning. The PCR purification kit (Qiagen, Germantown, MD) was used to purify DNA fragments. Primers and gRNAs used in this study are listed in Table 4. Plasmids were extracted using mini- or midi-kits (Qiagen). SnapGene software (www.snapgene.com) was used for primer design, identifications of DNA sequences and restriction sites, and generation of plasmid maps.

**Table 4:** Primers and gRNAs used in this study.

Primer Name	Sequence (5’-3’)
cas9-F	tacctcgcgaatgcatTTAAGAAATAATCTTCATCTAAAATATACTTC
cas9-R	atgcaggcctctgcaTCTTGCGGGATTACGAAATC
Fcas9eae	TGACCATGATTACGCCAAGC
Rcas9eae	AGTCGACTTAAGAAATAATCTTCATCTAAAATATACTTC (*SalI*)
R6KCMR	gattatttcttaagtcgactCTCGAGCGCTGAGATAGGTGCCTCAC (*XhoI*)
6KKCMF2	gtaatcatggtcactgcagaCCCGGGAAGCAGAAGGCCATCCTGAC (*SmaI*)
GRNA-F	TGACTGCAGCCTTGACAGCTAGCTCAGT (*PstI*)
GRNA-R	TGACCCGGGCAAAAAAAGCACCGACTCGGT (*SmaI*)
TetAR-F	TGAGTCGACCGTGTCGTCAGACCGTCTAC (*SalI*)
TetAR-R	CCACTCGAGCCACGATCCGCCCGATATAG (*XhoI*)
21Asi-F	CCAGCGATCGCTTAAGAAATAATCTTCATCTAAAATATACTTC (*AsiSI*)
21EcoNI-R	CCACCTGATTCAGGCATATGAATATCCTCCTTA (*EcoNI*)
gRNA*eae*	TGAAGCTTCCTTGACAGCTAGCTCAGTCCTAGGTATAATACTAGT*TCAGAGATCGCGACTGAAGC*GTTTTAGAGCTAGAAATAGCAAGTTAAAATAAGGCTAGTCCGTTATCAACTTGAAAAAGTGGCACCGAGTCGGTGCTTTTTTTGAAGCTTA (*HindIII*)
gRNA*ssaN*	TGAAGCTTCCTTGACAGCTAGCTCAGTCCTAGGTATAATACTAGT*CTGTGGCGAAGGGCAACGAG*GTTTTAGAGCTAGAAATAGCAAGTTAAAATAAGGCTAGTCCGTTATCAACTTGAAAAAGTGGCACCGAGTCGGTGCTTTTTTTGAAGCTTA
gRNA*bla*_CMY-2_	TGAAGCTTCCTTGACAGCTAGCTCAGTCCTAGGTATAATACTAGT*CAACGGCAGCGACAGCAAAG*GTTTTAGAGCTAGAAATAGCAAGTTAAAATAAGGCTAGTCCGTTATCAACTTGAAAAAGTGGCACCGAGTCGGTGCTTTTTTTGAAGCTTA

Note: The underline indicates the enzyme cutting site or gRNA targeting sequence.

To target *eae* of EPEC and EHEC, a 20-nt (TCAGAGATCGCGACTGAAGC) complementary region with the requisite TGG PAM matching genomic loci of *eae* was programmed directly into a CRISPR array containing a fused crRNA and tracrRNA as a single guide RNAeae. The de novo synthesized gRNA*eae* with a constitutive promoter pJ23119 [58] was cloned into the *HindIII* site of pUC57 (GenScript). Subsequently, *cas9* of pCas9 was cloned into the *XbaI*/*PstI* sites of the plasmid using primers cas9-F/cas9-R and Gibson assembly master mix (NEB) after the double digestions to obtain pWS10. To facilitate insertion of the CRISPR array into a desired genome, pWS20 was assembled (Gibson Assembly Tool, NEB) using a PCR fragment of pWS10 containing *cas9* and gRNA*eae*. This fragment was amplified using primers Fcas9eae and Rcas9eae, and a PCR fragment containing the R6K replicate origin and a *npt* that was amplified from pKD3 using primers R6KCMR and 6KKCMF2. To target *ssaN* of *S*. *enterica*, a synthesized gRNA*ssaN* containing 20-nt (CTGTGGCGAAGGGCAACGA) targeting sequence wad cloned into the *Sma*I/*Pst*I of pWS20 using primers GRNA-F/-R to generate pWS21 (Fig 1). Similarly, a synthesized gRNA*bla*_CMY-2_ containing the targeting 20-nt (TCAACGGCAGCGACAGCAAAG) was cloned into the *Sma*I/*Pst*I of pWS20 using primers GRNA-F/-R to generate pWS21B (S1 Fig). To introduce the CRISPR gRNA*eae* array into pRK24, a fragment of *tetAR* from pRK24 was amplified using primer TetAR-F/-R and cloned into the *Sal*I/*Xho*I sites of pWS20 to generate pWS20tet. The plasmid pWS20tet was conjugatively transferred into *E*. *coli* K12 (Rif^R^) containing pRK24 using *E*. *coli* S17-1. Transconjugants (Rif^R^, Amp^R^, Cm^R^) were selected and the plasmid pRK24-CRISPR resulted from homologous recombination was confirmed by PCR for the presence of the CRISPR array. To generate pTF16, a CRISPR-containing fragment was amplified from pWS21 (using primers 21AsiF/21EcoNI-R) was ligated to pBP136kan backbone generated by double digestions with *AsiSI*/*EcoN*. Similarly, to generate pTF18, a CRISPR-containing fragment that was amplified from pWS21B (using primers 21AsiF/21EcoNI-R) was ligated to the pBP136kan backbone following the double digestions with *Asi*SI/*Eco*NI (S1 Fig).

### Bacterial mating assays

Overnight cultures of donors and recipients were pelleted, washed, and resuspended in fresh LB broth. Donors and recipients were mixed in a volume ratio of 3:1 (150 μl:50 μl) for bi-parental or 2:1:1 (100 μl:50 μl:50 μl) for tri-parental matings. The mixtures were centrifuged at 13,000 g for 2 mins and cell pellets resuspended in 50 μl LB broth, spotted onto a sterile membrane filter (0.45-μm pore size, 25 mm in diameter, MilliporeSigma, Burlington, MA), overlaid on a LB agar, and incubated at 37°C for 6 h. The mating mixtures from the filters were resuspended in sterile phosphate-buffered saline (PBS), vortexed, serially diluted, and plated on appropriate selection media. Total viable cell counts of recipients and transconjugants were determined. Following a six h mating, the numbers of recipient cells were enumerated by plate count. The percentage of cell death (killing efficiency) of each strain was calculated using the equation (1—remaining number of the recipient cells / the initial number of the recipient cells) × 100%.

For the *bla*_CMY-2_ plasmid eliminating assay, donors, either strain EC100 containing pTF18 (CmR, *bla*_CMY-2_ targeting plasmid) or pTF16 (CmR, control plasmid), and the recipient strain *E*. *coli* C3b (NalR, CmR, AmpR) were grown separately in LB with the indicated antibiotics overnight. These the cultures were pelleted by centrifugation, washed and resuspended in fresh LB broth. Each donor and recipient *E*. *coli* C3b were mixed at a volume ratio of 5:1 (500 μl:100 μl). The mixtures were centrifuged at 13,000 g for 2 mins and cell pellets resuspended in 50 μl LB broth, spotted onto a 0.45-μm sterile membrane filter overlaid on a LB agar and incubated at 37°C for 18 h. The mating mixtures from the filters were resuspended in sterile PBS, and serially diluted and plated onto LB agar supplemented with Nal and incubated overnight at 37°C. From the countable plates (30 to 300 colonies), a total of 100 colonies were randomly toothpicked to fresh LB and LB-Amp agars. Total and Amp^S^ colonies were counted and the efficiency of plasmid elimination was calculated by dividing the number of Amp^S^ colonies by 100. All mating experiments were performed in triplicate.

### Murine experiments

A neonate murine model of EPEC colonization was used in this study [40]. All animal procedures were approved by the Institutional Animal Care and Use Committee of the University of Idaho under protocol #2017-31-v5. All appropriate steps were taken to assure animal welfare throughout this study to ameliorate suffering. Newborn mice infected with EPEC remained healthy throughout the two-week experiment and were euthanized at specific time points (below) to determine EPEC CFUs. At no time throughout the experiment did mice display markers associated with death or poor prognosis of quality of life or specific signs of suffering or distress.

Wild-type timed-pregnant female C57BL/6J mice were obtained from Jackson Laboratory (Bar Harbor, ME).The 3-day-old pups were separated from their mothers for 1 h. Each pup of three groups (n = 14, n = 15, and n = 14) was fed 1 x 10^5^ EPEC strain O55:H (Nal^R^). These inocula weregrown for 10 h in LB and washed with 1×PBS and concentrated to 1x10^5^ in 10 μl 20% sucrose. On day 1 and 2 post-infection (PI), the three groups of pups were treated with 1 x10^7^ CFU of *E*. *coli* MF1 pBP136kan (Rif^R^, Kan^R^) or *E*. *coli* MF1 pTF16 (Rif^R^, Cm^R^) in 10 μl 20% sucrose or PBS as shown in Table 2. On days 2, 3, 5, 7, 10, and 14 PI, the samples of small intestine and colon from at least 2 animals in each group were collected, homogenized in PBS, diluted and plated on agar plates containing the appropriate antibiotics for bacterial counts. For enrichment procedure, the samples were added to fresh LB broth and placed on a rotary shaker (150 rpm) and incubated at 37°C for 18 h, followed by serial dilution and plating on LB agar supplemented with Nal.

### Statistical analysis

GraphPad-Prism version 7.0 was used to determine statistical significance, and the following tests were employed. For mating-killing assays, a log normal distribution was assumed, and a Student’s t-test was performed. In the intestinal colonization study, differences in the number of bacteria in tissue samples between the treatment groups were compared by ANOVA repeated measures of analysis of variance using the log group geometric means of CFU/g tissue. P values indicate the following: *, P< 0.05; **, P< 0.001.

## Supporting information

S1 Fig Construction and maps of self-transmissible conjugative plasmids carrying CRISPR-Cas9 antimicrobials. (A) pWS10 was constructed to express a functional CRISPR-Cas9 system by inserting a gRNA*eae* and *cas9* into pUC57. gRNA*eae* was displayed with its secondary structure. Suicide plasmid pWS20 was constructed to contain B6Y origin, a *cat* gene from pKD3 and a *cas*9-gRNA*eae* fragment from pWS10. Self-transmissible plasmids pCRISPR-RK24, pBP136kan derivatives pTF16 and pTF18 were engineered to carry CRISPR-Cas9 systems that target *E*. *coli* and *Salmonella* pathogens and the *bla*_CMY-2_ antibiotic resistance gene. Plasmid maps were generated by the SnapGene Viewer. (B) The step-by-step construction of plasmids in A is diagrammed.
(TIF)
