# Supplementary figures and images for: Engineering conjugative CRISPR-Cas9 systems for the targeted control of enteric pathogens and antibiotic resistance
Source: PLoS One. 2023 Sep 12;18(9):e0291520. doi: 10.1371/journal.pone.0291520 (PMC10497133; doi:10.1371/journal.pone.0291520)

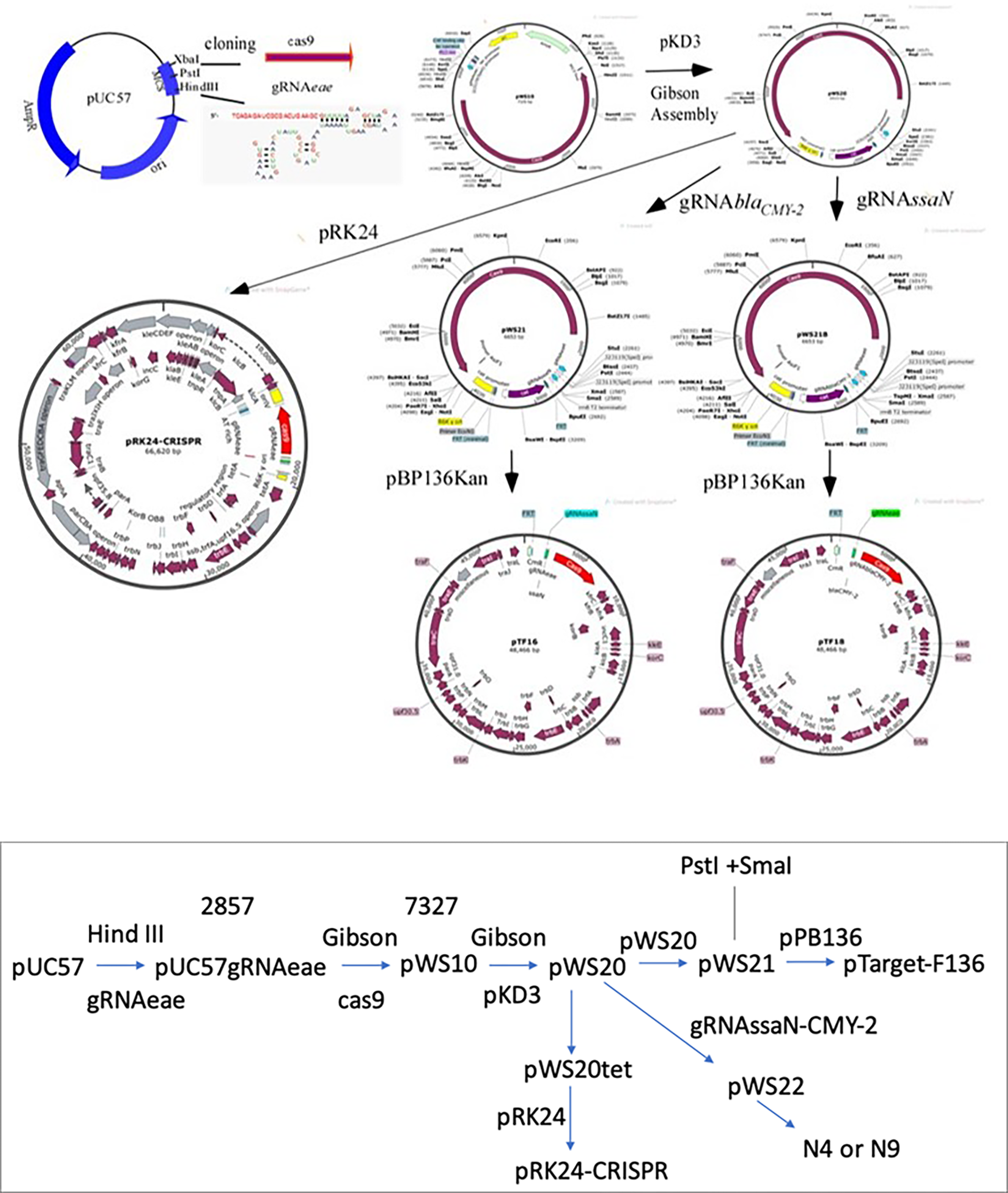

Supplement: S1 Fig — (A) pWS10 was constructed to express a functional CRISPR-Cas9 system by inserting a gRNAeae and cas9 into pUC57. gRNAeae was displayed with its secondary structure. Suicide plasmid pWS20 was constructed to contain B6Y origin, a cat gene from pKD3 and a cas9-gRNAeae fragment from pWS10. Self-transmissible plasmids pCRISPR-RK24, pBP136kan derivatives pTF16 and pTF18 were engineered to carry CRISPR-Cas9 systems that target E. coli and Salmonella pathogens and the blaCMY-2 antibiotic resistance gene. Plasmid maps were generated by the SnapGene Viewer. (B) The step-by-step construction of plasmids in A is diagrammed. (TIF) [file pone.0291520.s001.tif]
